# Who died of what in rural KwaZulu-Natal, South Africa: a cause of death analysis using InterVA-4

**DOI:** 10.3402/gha.v7.25496

**Published:** 2014-10-29

**Authors:** Joël Mossong, Peter Byass, Kobus Herbst

**Affiliations:** 1 Africa Centre for Health and Population Studies, University of KwaZulu-Natal, Somkhele KwaZulu-Natal, South Africa; 2 National Health Laboratory, Surveillance & Epidemiology of Infectious Diseases, Dudelange, Luxembourg; 3 Department of Public Health and Clinical Medicine, Umeå Centre for Global Health Research, Umeå University, Umeå, Sweden

**Keywords:** mortality, cause of death, demographic surveillance, South Africa, HIV/AIDS, tuberculosis, verbal autopsy

## Abstract

**Background:**

For public health purposes, it is important to see whether men and women in different age groups die of the same causes in South Africa.

**Objective:**

We explored sex- and age-specific patterns of causes of deaths in a rural demographic surveillance site in northern KwaZulu-Natal in South Africa over the period 2000–2011.

**Design:**

Deaths reported through the demographic surveillance were followed up by a verbal autopsy (VA) interview using a standardised questionnaire. Causes of death were assigned likelihoods using the publicly available tool InterVA-4. Cause-specific mortality fractions were determined by age and sex.

**Results:**

Over the study period, a total of 5,416 (47%) and 6,081 (53%) deaths were recorded in men and women, respectively. Major causes of death proportionally affecting more women than men were (all *p*<0.0001): human immunodeficiency virus/acquired immunodeficiency syndrome (HIV/AIDS) (20.1% vs. 13.6%), other and unspecified cardiac disease (5.9% vs. 3.2%), stroke (4.5% vs. 2.7%), reproductive neoplasms (1.7% vs. 0.4%), diabetes (2.4% vs. 1.2%), and breast neoplasms (0.4% vs. 0%). Major causes of deaths proportionally affecting more men than women were (all *p*<0.0001) assault (6.1% vs. 1.7%), pulmonary tuberculosis (34.5% vs. 30.2%), road traffic accidents (3.0% vs. 1.0%), intentional self-harm (1.3% vs. 0.3%), and respiratory neoplasms (2.5% vs. 1.5%). Causes of death due to communicable diseases predominated in all age groups except in older persons.

**Conclusions:**

While mortality during the 2000s was dominated by tuberculosis and HIV/AIDS, we found substantial sex-specific differences both for communicable and non-communicable causes of death, some which can be explained by a differing sex-specific age structure. InterVA-4 is likely to be a valuable tool for investigating causes of death patterns in other similar Southern African settings.

Despite the fact that the rationale for any successful health interventions should be informed by the cause of deaths (CODs) that are of the greatest importance locally, many developing countries lack this crucial information (1). Verbal autopsy (VA) has been shown as a useful tool in such settings to establish a probable COD by interviewing a close caregiver to give details on circumstances and symptoms leading up to the death event (2).

The Africa Centre for Health and Population Studies has hosted a longitudinal demographic surveillance system (DSS) in a rural area in South Africa which enables detailed analyses of CODs. Following the introduction in 2004 and massive scale-up thereafter of an antiretroviral treatment (ART) programme, mortality rates in adults and young children have declined significantly (3, 4). This decline has been essentially driven by a substantial decrease of deaths due to HIV/AIDS (5). In particular, the cause-specific mortality fraction (CSMF) of HIV-related causes declined from 56% in 2002 to 39% in 2009 and all-cause mortality rates have dropped by 33%. More recently, it was shown that life expectancy increased from 49.2 years in 2004 to 60.5 years in 2011 (6).

In 2012, WHO published a revised shortened VA questionnaire (7) which can be used in combination with a publicly available tool InterVA-4 to assign causes of death (8). In our and other rural contexts in Africa, the previous version InterVA-3 was shown to give substantial agreement with physician-coded causes of death (5, 9–11). A comparison between InterVA-3 and InterVA-4 using data from another demographic surveillance site in South Africa has shown continuity of interpretation (8).

The objective of this study is to further explore sex- and age-specific patterns of causes of deaths in our setting using the new tool InterVA-4 (8). We will analyse the major causes of deaths for men and women and focus particularly on sex and age disparities.

## Methods

### Study population

The DSS, which is located in the rural district of uMkhanyakude in northern KwaZulu-Natal in South Africa, has been running since January 2000. Roughly 11,000 households with approximately 60,000 resident and 30,000 non-resident members were visited twice a year to collect information on births, migrations, and deaths, as well as other demographic, health and socioeconomic data. The population is poor, has high HIV prevalence (12) and high incidence (13), although incidence is lower in areas with high ART coverage (14). The current analysis pertains to deaths of DSS residents reported to have occurred between 2000 and 2011.

All deaths reported by the demographic surveillance were followed up by a verbal autopsy (VA) interview conducted by a trained nurse with the closest available caregiver of the deceased. The VA interview was conducted at the earliest 3 months after the date of death, but on average 10 months after the death. It included an open narrative of the circumstances leading up to the death, a checklist of signs and symptoms, and a structured questionnaire substantially similar to the INDEPTH/WHO VA questionnaire (3). The check list and structured questionnaire were entered twice by different data capturers and validated by a third person.

### COD assignment using InterVA-4

For assigning causes of death, we downloaded the publicly available tool InterVA-4 (version 4.02) (15) and ran it on a standard Microsoft Windows 7 PC. InterVA-4 takes as input a text file where each row consists of death cases. The columns in the input file consist of one name and 245 indicator variables in a predefined order. Indicator is the terminology used by InterVA-4 to describe the whole range of items of information about the circumstances of a death, including basic background characteristics, details of any illness (signs and symptoms) leading to death, previous medical history, etc. All indicators are coded as ‘y’ for yes or ‘n’ for no. In addition, InterVA-4 requires the user to specify HIV and malaria prevalence, which were set on ‘high’ and ‘low’, respectively, for our setting. The questionnaire and check list data from the local database were extracted using Microsoft SQL and converted into the WHO 2012 standard VA format required by InterVA-4 (16). Of the 245 input indicators, 56 (23%) indicators were not available in the version of VA questionnaire used in this study.

InterVA-4 produced an output file containing for each death one to three causes of deaths and their respective likelihoods (8). One of the interesting features of InterVA-4 is that it explicitly allowed for multiple causes of death and enabled assignment of any residual likelihood for any individual case to be assigned as indeterminate. The residual likelihood is the fraction of the likelihood that could not be attributed to any particular COD, which can be considered as an individual-level expression of uncertainty in the COD assignment.

The full data set is available via the INDEPTH Network Data Repository (17).

### Statistical analysis

CSMFs were determined overall and by population subgroup (age or sex) by summing likelihoods across all one to three possible causes of death as determined by InterVA-4 and dividing by the sum of the likelihoods for all causes (18). For each COD, hypothesis testing for the difference of mortality fractions by sex was performed using the immediate form of two-sample test of proportion command ‘prtesti’ in Stata 12.1 (StataCorp, College Station, USA). Age-dependent mortality rates were calculated by dividing estimated number of deaths for a given cause by the person-years of observation. Hypothesis testing for the difference of mortality rates by sex in each age group was performed using the command ‘iri’ in Stata 12.1 (StataCorp, College Station, USA).

## Results

Following the demographic surveillance reports of 11,497 deaths between 2000 and 2011, 10,958 (95.3%) VA interviews were successfully conducted which were used as input data for InterVA-4. Approximately one in six deaths occurred before the age of 15 years, almost half of deaths occurred in adulthood between the ages of 15 and 49 years, and approximately one third in persons aged 50 years and older (Table 1). There were 5,416 (47%) and 6,081 (53%) deaths recorded in men and women, respectively. Deaths in males outnumbered those in women for neonates, infants, young children aged 1–4 years, and adults aged 50–64 years. Deaths in women outnumbered those in men for adults aged 15–49 years and persons aged 65 years or older.

**Table 1:** Distribution by age and sex of death counts, person years (PY) of observation and mortality rates of residents at Africa Centre demographic surveillance area in rural KwaZulu-Natal, 2000–2011

	Death counts		PY		Mortality rate (per 1,000)	
			
Sex	Male	Female	Male	Female	Male	Female
Neonate (<28 days)	96 (56%)	75 (44%)	840	818	114.3	91.7
Infant (1–11 months)	412 (52%)	388 (49%)	10,560	10,250	39.0	37.9
1–4 years	327 (53%)	290 (47%)	46,161	45,205	7.1	6.4
5–14 years	172 (50%)	169 (50%)	117,615	115,347	1.5	1.5
15–49 years	2,572 (47%)	2,945 (53%)	163,257	210,842	15.8	14.0
50–64 years	889 (53%)	781 (47%)	19,202	35,650	46.3	21.9
65+ years	948 (40%)	1,433 (60%)	11,122	28,038	85.2	51.1
Total	5,416 (47%)	6,081 (53%)	368,758	446,150	14.7	13.6

The following COD categories, which can potentially be assigned by InterVA-4, were not observed in our population: haemorrhagic fever, sickle cell with crisis, non-road transport accident, obstructed labour, and ruptured uterus. Overall, the fraction of deaths for which no COD could be determined was 10.6% and this did not vary significantly between men and women (10.7 and 10.5%, respectively, *p*>0.05). This fraction of unknown causes of death can be further divided into three categories: for 539 (4.7%) deaths no VA interview had been conducted, so obviously no information was available to assign a probable COD; for a further 192 (1.7%) deaths, although the VA interview had been conducted, the quality and breadth of information collected was insufficient or too unspecific for InterVA-4 to be able to assign any causes of death; and finally, the remaining 4.2% represented the residual likelihood of indeterminate causes of death.

Table 2 shows that CSMFs differed substantially between women and men in our population. Major causes of death proportionally affecting more women than men (ordered by decreasing difference and with a *p*<0.0001) were HIV/AIDS (20.1% vs. 13.6%), other and unspecified cardiac disease (5.9% vs. 3.2%), stroke (4.5% vs. 2.7%), reproductive neoplasms (1.7% vs. 0.4%), diabetes (2.4% vs. 1.2%), and breast neoplasms (0.4% vs. 0%). Major causes of deaths proportionally affecting more men than women (ordered by decreasing difference and with a *p*<0.0001) were assault (6.1% vs. 1.7%), pulmonary tuberculosis (34.5% vs. 30.2%), road traffic accidents (3.0% vs. 1.0%), intentional self-harm (1.3% vs. 0.3%), and respiratory neoplasms (2.5% vs. 1.5%).

**Table 2:** Cause-specific mortality fractions expressed as percentages in rural KwaZulu-Natal by sex

WHO VA cause of death code	Male (*N*=5,416)	Female (*N*=6,081)	Total (*N*=11,597)	F–M difference	*p*
01.01 Sepsis (non-obstetric)	0.04	0.03	0.04	−0.01	0.69
01.02 Acute resp infect incl. pneumonia	7.15	6.87	7.00	−0.27	0.56
01.03 HIV/AIDS related death	13.55	20.12	17.03	6.57	<0.0001
01.04 Diarrhoeal diseases	0.65	0.55	0.60	−0.11	0.46
01.05 Malaria	0.39	0.45	0.42	0.06	0.61
01.06 Measles	0.10	0.03	0.06	−0.06	0.18
01.07 Meningitis and encephalitis	0.79	1.00	0.90	0.21	0.23
01.08 & 10.05 Tetanus	0.01	0.00	0.01	−0.01	0.35
01.09 Pulmonary tuberculosis	34.52	30.15	32.21	−4.37	<0.0001
01.10 Pertussis	0.03	0.02	0.03	−0.01	0.71
01.99 Other and unspecified infect dis	0.22	0.10	0.16	−0.12	0.11
02.01 Oral neoplasms	0.12	0.09	0.10	−0.03	0.61
02.02 Digestive neoplasms	1.82	1.20	1.49	−0.62	0.006
02.03 Respiratory neoplasms	2.53	1.46	1.96	−1.07	<0.0001
02.04 Breast neoplasms	0.00	0.44	0.23		
02.05 & 02.06 Reproductive neoplasms MF	0.44	1.67	1.09	1.23	<0.0001
02.99 Other and unspecified neoplasms	1.11	0.76	0.92	−0.34	0.05
03.01 Severe anaemia	0.16	0.40	0.29	0.24	0.05
03.02 Severe malnutrition	0.43	0.45	0.44	0.02	0.86
03.03 Diabetes mellitus	1.21	2.35	1.81	1.13	<0.0001
04.01 Acute cardiac disease	0.64	0.38	0.50	−0.26	0.05
04.02 Stroke	2.74	4.51	3.68	1.77	<0.0001
04.99 Other and unspecified cardiac dis	3.23	5.94	4.66	2.71	<0.0001
05.01 Chronic obstructive pulmonary dis	1.18	1.85	1.54	0.67	0.004
05.02 Asthma	0.64	0.98	0.82	0.33	0.05
06.01 Acute abdomen	1.11	1.17	1.14	0.07	0.74
06.02 Liver cirrhosis	0.26	0.26	0.26	0.00	0.97
07.01 Renal failure	0.70	0.43	0.56	−0.27	0.05
08.01 Epilepsy	0.68	0.28	0.47	−0.40	0.002
09.01 Ectopic pregnancy		0.05	0.03		
09.02 Abortion-related death		0.03	0.02		
09.03 Pregnancy-induced hypertension		0.22	0.12		
09.04 Obstetric haemorrhage		0.13	0.07		
09.06 Pregnancy-related sepsis		0.09	0.05		
09.07 Anaemia of pregnancy		0.06	0.03		
09.99 Other and unspecified maternal CoD		0.05	0.03		
10.01 Prematurity	0.11	0.12	0.11	0.01	0.92
10.02 Birth asphyxia	0.42	0.25	0.33	−0.17	0.11
10.03 Neonatal pneumonia	0.70	0.41	0.54	−0.29	0.03
10.04 Neonatal sepsis	0.09	0.11	0.10	0.02	0.74
10.06 Congenital malformation	0.19	0.17	0.18	−0.02	0.82
10.99 Other and unspecified neonatal CoD	0.05	0.02	0.03	−0.03	0.32
12.01 Road traffic accident	3.00	1.05	1.97	−1.95	<0.0001
12.03 Accid fall	0.04	0.04	0.04	0.01	0.86
12.04 Accid drowning and submersion	0.38	0.18	0.28	−0.20	0.04
12.05 Accid expos to smoke fire & flame	0.21	0.34	0.28	0.13	0.20
12.06 Contact with venomous plant/animal	0.16	0.02	0.08	−0.14	0.008
12.07 Accid poisoning & noxious subs	0.04	0.06	0.05	0.01	0.77
12.08 Intentional self-harm	1.26	0.27	0.74	−0.98	<0.0001
12.09 Assault	6.08	1.66	3.74	−4.42	<0.0001
12.99 Other and unspecified external CoD	0.06	0.14	0.10	0.07	0.21
98 Other and unspecified NCD	0.04	0.08	0.06	0.05	0.31
99 Indeterminate	5.60	6.19	5.91	0.59	0.18
XX – VA not completed	5.10	4.32	4.69	−0.77	0.05

Table 3 shows that cause-specific morbidity fractions varied substantially by age. The fraction of deaths for which no cause could be determined (codes 99 and XX) was highest in children below the age of 5 years (14.8%) and in persons 65 years or older (14.3%) and lowest in adults aged 15–49 years (7.6%). In general, causes of death due to communicable diseases (WHO VA code chapter 01) predominated in all age groups except in persons aged 65 years and older. Acute respiratory infections including pneumonia appeared as one of the principal causes of death for children below 5 years of age, particularly neonates (56.9%) and infants (24.2%). HIV/AIDS was a major COD responsible for more than 10% of deaths in all age groups except neonates (0%) and persons older than 65 years (3.1%). Pulmonary tuberculosis was the predominant COD in children aged 5–14 years (26.6%) and in adults aged 15–64 years (48.8%). As expected, the mortality fractions of non-communicable causes of death of neoplasms, diabetes, stroke, and unspecified cardiac disease grew in importance beyond 50 years of age.

**Table 3:** Cause-specific mortality fractions expressed as percentages by age group

WHO VA cause of death code	Neonate (*N*=171)	Infant (*N*=800)	1–5 year (*N =*617)	5–14 year (*N=*341)	15–49 year (*N*=5,517)	50–64 year (*N*=1,670)	65+ year (*N*=2,381)	Total (*N*=11,497)
01.01 Sepsis (non-obstetric)		0.32	0.07	0.30			0.01	0.04
01.02 Acute resp infect incl. pneumonia		56.88	24.17	4.85	1.37	2.05	3.15	7.00
01.03 HIV/AIDS related death		19.66	37.62	16.24	22.25	12.62	3.13	17.03
01.04 Diarrhoeal diseases		3.68	2.16	0.51	0.09	0.11	0.72	0.60
01.05 Malaria		1.08	2.24	2.16	0.17	0.09	0.30	0.42
01.06 Measles		0.61	0.25	0.28				0.06
01.07 Meningitis and encephalitis	0.81	0.37	0.73	4.14	1.22	0.56	0.16	0.90
01.08 & 10.05 Tetanus				0.23				0.01
01.09 Pulmonary tuberculosis		0.71	5.82	26.60	48.77	33.35	13.58	32.21
01.10 Pertussis		0.18	0.28					0.03
01.99 Other and unspecified infect dis				1.03	0.14	0.06	0.26	0.16
02.01 Oral neoplasms					0.14	0.09	0.09	0.10
02.02 Digestive neoplasms					0.97	3.69	2.35	1.49
02.03 Respiratory neoplasms					1.08	3.52	4.50	1.96
02.04 Breast neoplasms					0.27	0.16	0.38	0.23
02.05 & 02.06 Reproductive neoplasms MF					0.73	1.60	2.46	1.09
02.99 Other and unspecified neoplasms			0.16	0.27	0.44	1.38	2.39	0.92
03.01 Severe anaemia				0.48	0.04	0.19	1.09	0.29
03.02 Severe malnutrition		0.73	3.80	0.80	0.11		0.54	0.44
03.03 Diabetes mellitus				0.14	0.33	2.99	5.86	1.81
04.01 Acute cardiac disease					0.25	1.29	0.93	0.50
04.02 Stroke				0.43	0.51	6.28	12.13	3.68
04.99 Other and unsp. cardiac disease				1.05	1.19	6.85	14.81	4.66
05.01 Chronic obstr. pulmonary disease				0.13	0.06	1.57	6.18	1.54
05.02 Asthma			0.32	1.61	0.21	1.37	2.21	0.82
06.01 Acute abdomen		0.41	0.16	2.66	0.71	1.78	2.08	1.14
06.02 Liver cirrhosis				0.32	0.18	0.47	0.46	0.26
07.01 Renal failure					0.27	0.68	1.60	0.56
08.01 Epilepsy		0.34	0.16	3.39	0.34	0.58	0.41	0.47
09.01 Ectopic pregnancy					0.06			0.03
09.02 Abortion-related death					0.03			0.02
09.03 Pregnancy-induced hypertension					0.24			0.12
09.04 Obstetric haemorrhage					0.15			0.07
09.06 Pregnancy-related sepsis					0.10			0.05
09.07 Anaemia of pregnancy					0.07			0.03
09.99 Other and unspecified maternal CoD					0.05			0.03
10.01 Prematurity	7.73							0.11
10.02 Birth asphyxia	22.35							0.33
10.03 Neonatal pneumonia	36.60							0.54
10.04 Neonatal sepsis	6.64							0.10
10.06 Congenital malformation	5.12	0.71	1.02					0.18
10.99 Other and unspecified neonatal CoD	2.29							0.03
12.01 Road traffic accident		0.12	1.44	9.92	2.57	1.32	0.77	1.97
12.03 Accid fall			0.16	0.19			0.12	0.04
12.04 Accid drowning and submersion	0.58		0.97	4.08	0.18	0.06		0.28
12.05 Accid expos to smoke fire & flame	0.58	0.37	0.80	0.29	0.08	0.17	0.62	0.28
12.06 Contact with venomous plant/animal		0.11	0.28	0.29	0.02	0.19	0.07	0.08
12.07 Accid poisoning & noxious subs			0.16	0.43	0.05		0.02	0.05
12.08 Intentional self-harm				1.91	1.24	0.36	0.15	0.74
12.09 Assault			1.61	2.64	5.70	3.29	1.76	3.74
12.99 Other and unspecified external CoD		0.05	0.15	0.72	0.03	0.13	0.17	0.10
98 Other and unspecified NCD					0.02	0.05	0.21	0.06
99 Indeterminate	17.31	5.67	7.51	6.35	3.41	6.60	10.01	5.91
XX – VA not completed		8.00	7.94	5.57	4.15	4.49	4.33	4.69

Empty cells represent no deaths except for maternally-related death codes starting with 09 that are only relevant for the 15–49 age group and neonatal death codes 10.01–10.04 and 10.99 that are only relevant for neonates.

The fraction of external causes of death was highest in children aged 5–14 years, principally from road traffic accidents (9.9%). Assault was the most common (5.7%) external COD of young adults aged 14–49 years, principally affecting men.

An age-stratified comparison of mortality rates between men and women for the most important causes of death (Table 4) shows that for most causes of death, mortality rates differ in almost all adult age groups. This is particularly the case for HIV/AIDS (except the age group of persons 65 years or older), pulmonary tuberculosis, digestive and respiratory neoplasms in persons 50 years or older, road traffic accidents, intentional self-harm, and assault. Age-dependent mortality rates were similar in all age groups between men and women for diabetes, stroke, and unspecified cardiac diseases.

**Table 4:** Comparison of mortality rates (per 100,000) between men and women for the most important causes of death in adults, stratified by age group

	15–49 year			50–64 year			65+ year		
			
	Male	Female	*p*	Male	Female	*p*	Male	Female	*p*
01.03 HIV/AIDS related death	226	407	<0.0001	522	310	0.0002	229	175	0.31
01.09 Pulmonary tuberculosis	783	669	<0.000	1,741	625	<0.0001	1,730	467	<0.0001
02.02 Digestive neoplasms	20	10	0.02	191	70	0.0001	267	94	0.0001
02.03 Respiratory neoplasms	19	13	0.17	226	43	<0.0001	559	161	<0.0001
02.05 & 02.06 Reproductive neoplasms MF	2	17	<0.0001	12	68	0.002	162	145	0.71
03.03 Diabetes mellitus	7	3	0.15	74	101	0.30	360	355	0.95
04.02 Stroke	9	6	0.30	256	156	0.01	748	733	0.90
04.99 Other and unspecified cardiac disease	12	22	0.02	225	200	0.54	1,004	859	0.17
12.01 Road traffic accident	70	13	<0.0001	90	13	0.0001	94	28	0.02
12.08 Intentional self-harm	34	6	<0.0001	31	0	0.002	18	6	0.39
12.09 Assault	162	24	<0.0001	182	56	<0.0001	180	78	0.009

## Discussion

This is the first report on causes of death in rural KwaZulu-Natal based on the standardised 2012 WHO VA instrument and InterVA-4 tool. The results presented here are in line with previously published work using causes of death based on physician-coded diagnoses or InterVA-3. As reported previously, mortality in our setting during the 2000s was largely dominated by HIV/AIDS and tuberculosis (3, 5, 19, 20), although HIV-related mortality rates have declined substantially in recent years (3, 5), resulting in a dramatic increase in life expectancy (6). Our results are also in broad agreement with the Agincourt DSS in a different rural area in South Africa (10), although the fraction of undetermined causes of deaths are substantially lower in our setting (8). This suggests that the InterVA-4 tool for determining causes of death in combination with the 2012 VA instrument is appropriate for wider use in other Southern African settings.

One finding which warrants further investigation is the differing sex and age patterns of causes of death due to HIV/AIDS and tuberculosis. One the one hand, the observed sex-specific difference could be due to differing prevalence patterns of HIV/AIDS and tuberculosis between sexes. In 2010 in our setting, women were significantly more likely to be infected with HIV than men (27.6% vs. 16.2%) (12), which might explain the higher fraction of deaths due to HIV/AIDS observed in women. As the age group most affected by HIV/AIDS were women between the ages of 25 and 49 years, this could be a plausible explanation why we observed more deaths in women than men for the age group of 15–49 years. Similarly, it is known that in many settings in the world, notification rates of tuberculosis are higher in men than women, due to some extent by higher prevalence of cigarette smoking (21). This would explain the higher proportion of deaths due to tuberculosis in men compared to women. The high proportion of deaths attributed to tuberculosis in children aged 5–14 years is likely to be related to the high prevalence of HIV/AIDS in this population. Given the considerable overlap in mortality from HIV infection and tuberculosis (22), much previous work has therefore often combined the two categories of HIV and tuberculosis (3, 5). For future work, it would be interesting to investigate why InterVA-4 produces a higher fraction of deaths due to tuberculosis in comparison to HIV/AIDS, and whether the known uncertainty in COD for this important group is also reflected in the likelihoods.

An earlier analysis of causes of child mortality also highlighted the importance of lower respiratory tract infections as a COD in children under 1 year of age, which corresponds to our finding of a high CSMF of acute respiratory infections including pneumonia (20). A recent review indicated that 43% of childhood deaths occurred due to pneumonia in sub-Saharan Africa in 2011 (23).

Our study has several limitations. First, overall in our study, approximately 10% of all deaths could not be attributed to any of the causes of death, either because no VA interview was conducted or because the information provided by the informant was too unspecific for InterVA-4 to be able to assign a COD. Interestingly this fraction of unknown causes of death was found to vary by age. For young children between the age of 1 month and 5 years, we observed a higher fraction of VA interviews not being completed. This lower VA completion rate could be due to the parent not wanting to share information, because they are still traumatised by their loss. Although VA interviews in our setting are conducted at least 3 months after the date of death, a longer period might be considered for childhood deaths to give parents more time for the bereavement process. For neonates and persons older than 65 years, the fraction of indeterminate deaths (code 99) is higher than in other age groups and this could be an indication that in these age groups, assigning a COD is more difficult, either because informants do not have enough information or deaths occur with less discernible symptoms.

Another limitation is that although our overall sample size is large, many causes of deaths occurred infrequently: for 30 causes of death, the CSMF was less than 0.5% such that our study could be underpowered to assess sex-specific differences for these cause categories. Moreover, because of the large number of statistical tests conducted, we acknowledge that there is a potential risk of type I errors. Nevertheless, because our focus was on causes of death differences with *p*-values below 0.0001, a conservative Bonferroni correction for 50 tests would still make these sex-specific differences significant at level *p*<0.005.

As far as non-communicable diseases are concerned, we found that CSMFs of stroke and diabetes were generally higher in women than in men. As the age-stratified analysis has shown, this can be explained by the longer life expectancy we observe for women in our setting (6), such that many more women than men attain an age where these underlying conditions can cause death. It is generally assumed that with the epidemiological transition occurring in low- and middle-income countries like South Africa, that non-communicable diseases will grow in importance and that this will eventually reflect in COD patterns. So far, there is little sign in our mortality data of such an increasing trend (data not shown), but it is obviously an area of interest and of global public health concern in the years to come.

## Conclusions

Mortality during the 2000s in our DSS continued to be dominated by tuberculosis and HIV/AIDS. For certain communicable and non-communicable causes of death, we identified sex-specific differences of mortality fractions, some of which can be explained by the fact that there is a higher proportion of older women than men living in the DSS. InterVA-4 is a valuable tool for investigating causes of death patterns in Southern African settings.
